# Endurance exercise training suppresses myostatin upregulation and nuclear factor-kappa B activation in a mouse model of Parkinson’s disease

**DOI:** 10.14202/vetworld.2022.383-389

**Published:** 2022-02-18

**Authors:** Nour S. Erekat, Muhammed D. Al-Jarrah

**Affiliations:** 1 Department of Anatomy, Faculty of Medicine, Jordan University of Science and Technology, Irbid 22110, Jordan; 2 Department of Rehabilitation Sciences, Faculty of Applied Medical Sciences, Irbid 22110, Jordan

**Keywords:** gastrocnemius, immunohistochemistry, myostatin, nuclear factor kappa B, Parkinson’s disease

## Abstract

**Background and Aim:**

Muscle atrophy is common in Parkinson’s disease (PD). Although myostatin has been implicated in muscle atrophy, its expression in PD skeletal muscle has not been investigated. Therefore, this study aimed to elucidate the influence of PD induction and exercise training on myostatin expression in the gastrocnemius skeletal muscle.

**Materials and Methods:**

Thirty albino mice were randomly selected and separated into three groups of 10 mice each: Sedentary control, sedentary PD (SPD), and exercised PD (EPD). 1-Methyl-4-phenyl-1,2,3,6-tetrahydropyridine and probenecid were used to induce chronic parkinsonism in the PD groups. Immunohistochemistry was used to investigate the expression of myostatin and nuclear factor kappa B (NF-kB) in gastrocnemius muscles of all three groups.

**Results:**

Myostatin expression and NF-kB nuclear localization, indicative of its activation, were significantly (p<0.01) higher in gastrocnemius skeletal muscle in the SPD group than in the control and EPD groups. Concomitantly, the average cross-sectional area of gastrocnemius muscle fibers in the SPD albino mice was significantly smaller (p<0.01) than in the control and EPD groups, indicating muscle atrophy.

**Conclusion:**

The present data are the first to indicate a correlation between PD induction and myostatin overexpression and NF-kB activation in the gastrocnemius muscle, potentially promoting the muscle atrophy commonly seen in PD. Additionally, the current data are the first to indicate the beneficial effects of exercise training on PD-associated myostatin overexpression, NF-κB activation, and muscle atrophy. Thus, our data are the first to suggest that myostatin and NF-κB might be regarded as potential therapeutic targets in an attempt to ameliorate skeletal muscle abnormalities commonly observed in PD.

## Introduction

Parkinson’s disease (PD) is the second most common neurodegenerative disease in the elderly [1-4]. It occurs due to a substantial decrease in dopamine secretion caused by the degeneration of dopaminergic neurons in the substantia nigra pars compacta [5-9]. Skeletal muscle atrophy has been reported in PD [1-4]. Overexpression of myostatin, a negative regulator of skeletal muscle mass, has been shown in atrophied skeletal muscles [10]. In addition, the systemic administration of myostatin results in muscle atrophy in mice. Consequently, myostatin overexpression has been speculated to contribute to muscle atrophy, which is associated with aging and many pathological conditions [11-13]. Indeed, myostatin overexpression has been reported in skeletal muscles, where it is associated with their mass loss in a rat model of chronic heart failure (CHF). Myostatin overexpression is reportedly induced by the pro-inflammatory cytokine tumor necrosis factor-alpha (TNF-α) through the activation of nuclear factor kappa B (NF-κB) [14]. In contrast, exercise training significantly suppresses myostatin overexpression, suggesting that the beneficial effects of exercise training in skeletal muscle of rat CHF model might be mediated by the suppression of inflammatory cytokines, resulting in reduced myostatin levels [14].

In addition, local expression of TNF-α and activation of NF-κB were significantly increased in the skeletal muscles of mice following PD induction using 1-methyl-4-phenyl-1,2,3,6-tetrahydropyridine (MPTP) [15]. Furthermore, exercise training has been reported to benefit PD patients [16,17]. However, alterations in myostatin expression in PD and the effects of exercise training on myostatin levels have not yet been elucidated. Therefore, we hypothesized that the induction of PD and exercise training might be associated with alterations in the expression of myostatin and activation of NF-κB in gastrocnemius skeletal muscle, indicating that they may have potential pathological roles in the development and progression of skeletal muscle atrophy observed in PD. In consequence, they might be regarded as potential therapeutic targets in attempts to ameliorate skeletal muscle atrophy seen in PD patients.

Therefore, this study aimed to assess myostatin expression and the impact of exercise training on myostatin and NF-κB levels in the gastrocnemius muscle of an established mouse model of PD.

## Materials and Methods

### Ethical approval

Animal protocols were approved by the Institutional Animal Care and Use Committee of Jordan University of Science and Technology (approval no. 16-3-3-347).

### Study period and location

The study was conducted from May 2017 to May 2021, at the Department of Anatomy, Faculty of Medicine, Jordan University of Science and Technology.

### Animals

This study used thirty albino mice provided by the Animal House, Jordan University of Science and Technology, Jordan. They were allocated to three groups (n=10 animals/group): Sedentary control (SC), sedentary PD (SPD), and exercised PD (EPD). The albino mice were housed in separate cages under the same conditions. PD was induced in the PD groups by administering a subcutaneous injection of MPTP hydrochloride (25 mg/kg) (Selleck Chemicals, Pittsburgh, PA, USA) and an intraperitoneal injection of probenecid (250 mg/kg, in Tris-HCl buffer) (Selleck Chemicals) every 3 and half days, for 5 weeks, using a previously described protocol [18-20]. Simultaneously, intraperitoneal injections of saline (25 mg/kg) (Santa Cruz Biotechnology, Santa Cruz, CA, USA) were administered to the control mice. Four weeks after the last subcutaneous injection of MPTP and intraperitoneal injection of probenecid all mice in the 3 groups were sacrificed.

### Exercise protocol

All 30 albino mice were transferred to the training area daily at 9:00 a.m. to be subjected to the same environment. However, only those in the EPD group were trained according to a previously described exercise training protocol, which had been reported to afford sufficient systemic and cellular adaptations [21-25]. In brief, a custom treadmill (Columbus Instruments, Columbus, OH, USA) with 10 separate lanes was used to make the EPD mice run, at a speed of 18 m/min for 40 min/day for 5 days/week for 4 weeks.

### Immunohistochemistry of myostatin and NF-κB in the skeletal muscle

Immunohistochemistry for myostatin and NF-κB was performed according to a previously described protocol [26-29]. In brief, 4 μm thick sections of paraffin-embedded gastrocnemius skeletal muscle were prepared, deparaffinized, and rehydrated. Next, 3% hydrogen peroxide in methanol was used to block endogenous peroxidase activity. Phosphate-buffered saline (PBS, pH 7.2) was used to wash the sections before and after incubating some of them with myostatin antibody (Santa Cruz Biotechnology, Santa Cruz, CA, USA) and incubating others with anti-NF-κB antibody (Santa Cruz Biotechnology), using the dilutions suggested by the manufacturers. The sections were then incubated with a biotinylated secondary antibody (LSAB kit, Dako, Carpinteria, CA, USA) and washed with PBS before being successively incubated with streptavidin horseradish peroxidase (LSAB kit, Dako). They were then rinsed with PBS and treated with 3,3′-diaminobenzidine substrate (Santa Cruz Biotechnology) until the desired color intensity was detected. Finally, the sections were washed with tap water to stop the further reaction. Hematoxylin (Santa Cruz Biotechnology) was used to counterstain the sections. Negative control slides were processed in the absence of a primary antibody. The sections were viewed at 40x under a light microscope (BEL Engineering Company, Italy).

### Muscle loss assessment

Serial transverse 4 μm thick sections of paraffin-embedded gastrocnemius muscles were prepared, deparaffinized, rehydrated, and stained with hematoxylin and eosin (Santa Cruz Biotechnology). Five slides from each animal in each of the three groups were examined under light microscope at 40x (BEL Engineering Company, Italy), and five random areas in each section were photographed using a digital camera (BEL Engineering Company). Cross-sectional areas of the muscle fibers were measured using ImageJ software (US National Institutes of Health, Bethesda, Maryland, USA), as previously suggested [19,30,31]. Subsequently, the average muscle fiber cross-sectional area was calculated for each animal in each group.

### Data collection and analysis

Five slides from each mouse in every group were examined by light microscopy. Five random areas from each section were photographed with a digital camera (BEL Engineering Company) and successively inspected for myostatin expression and NF-κB activation in the gastrocnemius skeletal muscle. Next, the entire positively stained pixel area relative to the entire pixel area was calculated in every photographed area in each section, using Adobe Photoshop software (Adobe Systems, San Jose, CA, USA), as previously described [15,20]. Subsequently, the average of the positively stained pixel regions relative to the entire pixel area was computed for each animal in every group.

### Statistical analysis

Averages of myostatin expression, NF-κB activation, and muscle fiber cross-sectional areas in the skeletal muscles were calculated for each group and statistically compared among the three groups (n=10 animals/group) by one-way analysis of variance using Statistical Package for the Social Sciences (SPSS) software (version 19.0; SPSS Inc., Chicago, IL, USA). Variations in the averages of myostatin expression, NF-κB activation, and muscle fiber cross-sectional area were considered statistically significant at p<0.05.

## Results

**Figure-1:**
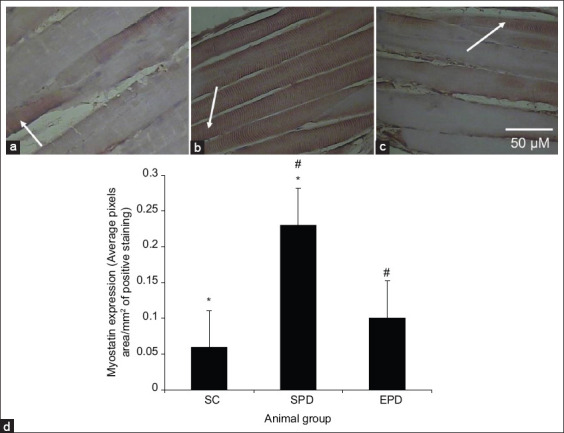
Immunohistochemical staining of myostatin in 4 μm thick sections of paraffin-embedded gastrocnemius muscle. (a) Control, (b) SPD, (c) EPD scale bar shown in (c) applies to all images. Myostatin immunostaining can be observed in the control group. In contrast, although myostatin immunoreactivity was very strong in the SPD group (at the tips of the arrows), it was significantly decreased following endurance exercise training in the EPD group. (d) The level of myostatin expression was significantly higher in the SPD group than in the control group (p<0.01, *) but it was significantly lower (p<0.01, #) in the EPD group. SC=Sedentary control, SPD=Sedentary Parkinson’s disease, EPD=Exercised Parkinson’s disease. Scale bar = 50 μM.

Myostatin immunoreactivity could be detected in the gastrocnemius muscle sections from the control group (Figure-1a). In contrast, myostatin immunoreactivity was very abundant in the gastrocnemius muscle sections from the SPD group (Figure-1b), but not in the EPD group following endurance exercise training (Figure-1c).

Myostatin expression was significantly (p<0.01) increased in the gastrocnemius muscle following PD induction by MPTP/p treatment compared with that in control (Figure-1d). In contrast, it was significantly decreased (p<0.01) in the gastrocnemius muscle of the EPD group following endurance exercise training compared to that in the SPD group (Figure-1d).

**Figure-2:**
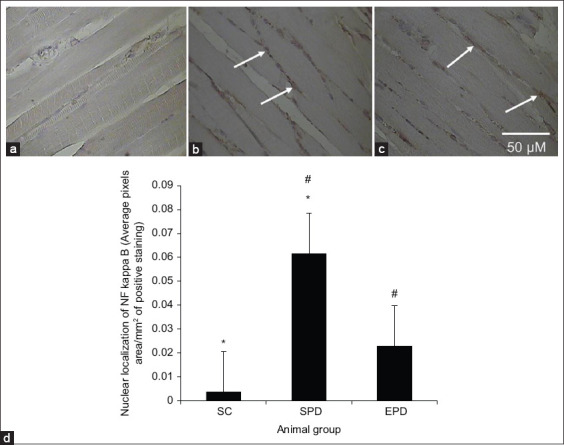
Immunohistochemical staining of nuclear factor kappa B (NF-κB) in 4 µm thick sections of paraffin-embedded gastrocnemius muscle. (a) Control, (b) SPD, (c) EPD scale bar shown in (c) applies to all images. Nuclear localization of NF-κB was barely detectable in gastrocnemius muscle sections from the control. In contrast, although NF-κB nuclear localization was very strong in the SPD group (at the tips of the arrows), it was significantly decreased following endurance exercise training in the EPD group. (d) Nuclear localization of NF-κB was significantly higher in the SPD group than in the control group (p<0.01, *), but was significantly decreased (p<0.01, #) in the EPD group following endurance exercise training. SC=Sedentary control, SPD=Sedentary Parkinson’s disease, EPD=Exercised Parkinson’s disease. Scale bar = 50 µM.

Similarly, NF-κB activation, as indicated by its nuclear localization, was barely detectable in gastrocnemius muscle sections from the control group (Figure-2a). In contrast, immunohistochemistry revealed abundant nuclear localization of NF-κB, suggestive of its activation, in the SPD group (Figure-2b), but not in the EPD group after chronic exercise training (Figure-2c).

NF-κB activation was significantly (p<0.01) elevated in the gastrocnemius muscle after PD induction by MPTP/p treatment compared with that in the control gastrocnemius muscle (Figure-2d). In contrast, NF-κB activation was significantly (p<0.01) reduced in the gastrocnemius muscle of the EPD group after endurance exercise training when compared with that in the gastrocnemius muscle of the SPD group (Figure-2d).

**Figure-3:**
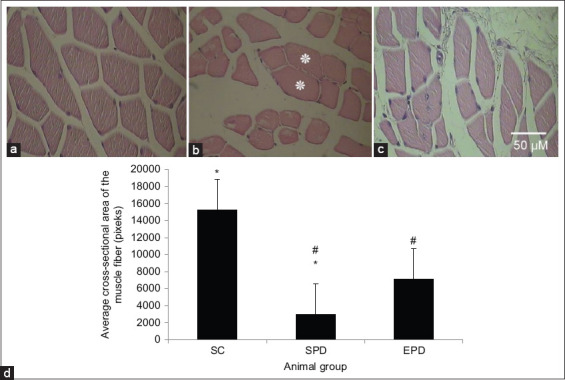
Morphological features displayed by gastrocnemius muscle fibers after PD induction by methyl-4-phenyl-1,2,3,6-tetrahydropyridine and probenecid treatment. Transverse hematoxylin and eosin-stained 4 µm thick sections of paraffin-embedded gastrocnemius muscle from control (a), SPD (b), and EPD (c) groups. Magnification bar displayed in (c) applies to all images. Atrophic muscle fibers and relatively normally sized muscle fibers (*) can be seen in gastrocnemius muscle of SPD mice, whereas polygonal normally looking muscle fibers can be seen in the gastrocnemius muscle of control mice. In contrast, muscle fibers from EPD mice appeared larger than those from SPD mice. (d) The average cross-sectional area of muscle fibers evaluated in transverse 4 µm thick sections of paraffin-embedded gastrocnemius muscles. The average cross-sectional area of the muscle fibers was significantly reduced (p<0.01, *) in the SPD group compared to that in the control group. In contrast, the average cross-sectional area of muscle fibers in the EPD group was significantly (p<0.01, #) higher than that in the SPD group. SC=Sedentary control, SPD=Sedentary Parkinson’s disease, EPD=Exercised Parkinson’s disease. Scale bar = 50 µM.

Gastrocnemius muscles from the control group appeared polygonal in shape (Figure-3a). However, gastrocnemius muscle fibers from the SPD group appeared atrophic and displayed smaller sizes and angular profiles (Figure-3b). In addition, some muscle fibers displaying a relatively normal size were observed among the atrophic muscle fibers from the SPD gastrocnemius muscle (Figure-3b). Muscle atrophy was assessed as a decline in the cross-sectional areas of muscle fibers (Figure-3d). The average cross-sectional area of gastrocnemius muscle fibers was significantly (p<0.01) reduced following the induction of PD in the SPD group (Figure-3d). However, it was increased in the gastrocnemius muscle from the EPD group subsequent to chronic exercise training (Figure-3c and d).

## Discussion

The current study is the first to elucidate the influence of PD induction on myostatin expression in gastrocnemius skeletal muscle. The resultant analysis revealed myostatin overexpression in the gastrocnemius muscle following the induction of chronic parkinsonism by MPTP/p treatment. In addition, PD-associated myostatin overexpression and NF-κB activation were attenuated in the gastrocnemius muscle after endurance exercise training.

MPTP treatment in conjunction with the adjuvant probenecid has become a useful technique for inducing chronic parkinsonism in mice [18,32]. Three weeks following MPTP/p treatment, only 20-30% of the total striatal dopamine levels and approximately one-third of the total dopaminergic neurons were found [33]. Concurrently, substantial motor dysfunction and impaired performance began as early as 3 days after MPTP/p treatment [18]. Motor dysfunction has been linked to skeletal muscle abnormalities in PD [34]. We have shown skeletal muscle atrophy manifested by a considerable reduction in the cross-sectional area of the PD gastrocnemius muscle in MPTP/p-treated albino mice 4 weeks after treatment [19]. As muscle atrophy has been shown to be induced by upregulated myostatin in aging and various pathological conditions [13,35,36], we sought to examine alterations in the expression of myostatin and NF-κB, as a potential mechanism underlying the muscle atrophy seen in PD, 4 weeks after MPTP/p treatment.

Myostatin, a transforming growth factor-b ligand that negatively regulates muscle growth [11,37], is a catabolic factor that increases under conditions that lead to muscle wasting [38] and is pathologically upregulated in skeletal and cardiac muscles [39,40]. TNF-α stimulates myostatin expression through the NF-κB-involved pathway in heart failure [10,13]. We have previously shown TNF-α upregulation and NF-κB activation in gastrocnemius skeletal muscle 4 weeks after PD induction by MPTP/p treatment [15]. The present results are consistent with this as they reveal myostatin upregulation (Figure-1b and d) along with NF-κB activation (Figure-2b and d) in the gastrocnemius muscle after PD induction [15].

Mitochondrial abnormalities have been reported in the skeletal muscle of PD patients [34,41,42]. They have been shown to cause overproduction of reactive oxygen species (ROS) and subsequent oxidative stress [43,44]. Increased ROS production has been demonstrated to induce TNF-α expression, which subsequently stimulates myostatin production through NF-κB [10,45]. Moreover, myostatin stimulates NF-κB signaling, which is correlated with TNF-α production and increases ROS formation [10]. Consequently, a feed-forward loop that further augments myostatin expression through NF-κB has been suggested [10].

Thus, it could be inferred that the significantly upregulated myostatin and NF-κB activation in the gastrocnemius muscle, as revealed by our results, might have contributed to the muscle atrophy indicated by the significant reduction in the average cross-sectional area of the SPD skeletal muscle (Figure-3b and d).

Exercise has been reported to have beneficial effects in patients with PD, including the amelioration of their motor symptoms [46]. To examine the potential mechanism by which exercise improves PD skeletal muscle abnormalities, we examined the effect of endurance exercise training on myostatin expression in the skeletal muscles of an established mouse model of PD.

Exercise training has been shown to enhance mitochondrial biogenesis by promoting mitochondrial function [47,48]. This leads to decreased formation of ROS and reduced oxidative stress, leading to decreased production of pro-inflammatory cytokines such as TNF-α upregulation [49,50]. PD-associated TNF-α upregulation has been shown to be suppressed in the gastrocnemius skeletal muscle following endurance exercise training [51], and attenuated TNF-α upregulation has been shown to decrease NF-κB activation [52]. Consistent with this, our results have revealed decreased NF-κB activation in the gastrocnemius muscle of EPD mice following endurance exercise training (Figure-2c and d). Reduced NF-κB activation after exercise training has been shown to suppress myostatin overexpression in CHF skeletal muscles and concomitantly reduce muscle atrophy in heart failure [14,53]. Consistent with this, our findings revealed suppressed myostatin overexpression (Figure-1c and d) correlated with reduced muscle atrophy, which was indicated by increased cross-sectional area (Figure-3c and d), in the EPD gastrocnemius muscle following endurance exercise training.

Therefore, we deduce that exercise training might have reduced ROS formation and led to attenuated TNF-α upregulation, which might have suppressed NF-κB activation, resulting in suppressed myostatin overexpression in the PD gastrocnemius skeletal muscle.

## Conclusion

The present data are the first to indicate the impact of PD and exercise training on myostatin expression and NF-κB activation in skeletal muscles. In summary, myostatin expression and NF-κB activation were increased and concomitant muscle atrophy was seen as indicated by a significant reduction in the average cross-sectional area of muscle fibers in the SPD gastrocnemius muscle. However, in EPD skeletal muscles following endurance exercise training, myostatin overexpression and NF-κB activation were attenuated, with concurrent reduced muscle atrophy, indicated by an increased average cross-sectional area of muscle fibers. Therefore, myostatin and NF-κB are probably implicated in skeletal muscle atrophy seen in PD and could be potentially targeted therapeutically to ameliorate skeletal muscle abnormalities characterizing PD.

## Authors’ Contributions

NSE: Designed the study, collected the data, performed the data analysis and interpretation, drafted the manuscript, and revised the manuscript. MDA: Collected the data, analyzed and interpreted the data, and revised the manuscript. Both authors read and approved the final manuscript.
